# Exploring Nurses’ Intention to Use a Computerized Platform in the Resuscitation Unit: Development and Validation of a Questionnaire Based on the Theory of Planned Behavior

**DOI:** 10.2196/ijmr.2150

**Published:** 2012-09-13

**Authors:** Christian Malo, Xavier Neveu, Patrick Michel Archambault, Marcel Émond, Marie-Pierre Gagnon

**Affiliations:** 1 Département de médecine familiale et de médecine d’urgenceFaculté de MédecineUniversité LavalQuébec, QCCanada; 2 Traumatologie – Urgence – Soins intensifsCentre de recherche FRQS du Centre hospitalier affilié universitaire de QuébecQuébec, QCCanada; 3 Centre de santé et de services sociaux Alphonse-Desjardins (CHAU de Lévis)Département d'urgenceQuébec, QCCanada; 4 Centre de recherche du Centre hospitalier universitaire de Québec (CRCHUQ)Québec, QCCanada

**Keywords:** Primary care nurses, adoption of new behavior, intention, theory of planned behaviour, emergency department, trauma care, electronic health record, clinical decision support system

## Abstract

**Background:**

In emergency department resuscitation units, writing down information related to interventions, physical examination, vital signs, investigations, and treatments ordered is a crucial task carried out by nurses. To facilitate this task, a team composed of emergency physicians, nurses, and one computer engineer created a novel electronic platform equipped with a tactile screen that allows systematic collection of critical data. This electronic platform also has medical software (ReaScribe+) that functions as an electronic medical record and a clinical decision support system.

**Objective:**

To develop and validate a questionnaire that can help evaluate nurses’ intention to use a novel computerized platform in an emergency department resuscitation unit, based on Ajzen’s theory of planned behavior (TPB).

**Methods:**

The sample for this study was composed of 87 nurses who worked in the resuscitation unit of a tertiary trauma center. We held three focus groups with nurses working in the resuscitation unit to identify the salient modal beliefs regarding their intended use of a new electronic medical charting system for the care of trauma patients. The system included a clinical decision support tool. We developed a questionnaire in which salient modal beliefs were used as items to evaluate the TPB constructs. We also added 13 questions to evaluate nurses’ computer literacy. The final questionnaire was composed of 46 questions to be answered on a 7-point Likert scale. All nurses in the resuscitation unit and present during a regular work shift were individually contacted by the principal investigator or a research assistant (phase 1). A subsample of the nurses who completed the questionnaire was invited to complete it a second time 2 weeks later (phase 2).

**Results:**

In phase 1, we received 62 of the 70 questionnaires administered (89% response rate). Of the 27 questionnaires administered in phase 2 (retest phase), 25 were completed (93% response rate). The questionnaire showed very good internal consistency, as Cronbach alpha was higher than .7 for all constructs. Temporal stability was acceptable with intraclass correlations between .41 and .66. The intention to use the electronic platform to chart the resuscitation of trauma patients was very high among the respondents. In the logistic regression model, the only construct that predicted nurses’ intention to adopt the computerized platform was the professional norm (odds ratio 3.31, 95% confidence interval 1.41–7.78).

**Conclusions:**

We developed and validated a questionnaire that can now be used in other emergency departments prior to implementation of the computerized platform. The intention to adopt was very high among the respondents, which suggests that the implementation of this innovation could be successful at our institution.

## Introduction

In the resuscitation room, particularly for a patient with multiple injuries, the documentation of critical patient data is a challenge for nurses. Nurses are required to write down information concerning interventions, physical examinations, vital signs, investigations, and treatments that are ordered. Given the stress in the resuscitation environment, handwriting all these data may result in information loss or errors in the documentation. For example, in the case of a tertiary trauma center, Kind et al found that 20% of vital signs were absent from a patient’s chart in the 15 minutes following endotracheal intubation [1]. To minimize these difficulties, a team composed of clinicians (emergency physicians and nurses) and a computer engineer created an electronic platform equipped with a tactile screen that allows for the systematic collection of critical data in the resuscitation unit. The electronic platform is an electronic medical charting system that integrates a clinical decision support tool (ReaScribe+; ReaEvolution inc, Québec, Canada) for the care of trauma patients. It allows automatic recording of vital signs, charting of administered medication, intravenous line use, and general information related to patient care. Furthermore, it informs the radiology department of investigations required immediately.

The impact of a positive or negative attitude on the part of staff in relation to the integration of new technology at work has been fully documented [2-5]. Moreover, in the last 30 years, numerous studies have evaluated nurses’ attitudes toward computer use [6,7]. In a systematic review, Huryk found that several factors influence nurses’ attitudes toward the implementation of new technologies in health care. Nurses between 30 and 39 years of age, those 60 years of age and over, and those with a higher level of computer experience are more likely to adopt a positive attitude toward electronic patient records. Older nurses and those with more computer and nursing experience, who hold higher staff positions, or have higher levels of education had more positive attitudes toward clinical management systems [7]. Furthermore, the provision of adequate training and support during implementation and the inclusion of interested nurses on the implementation committee are all actions that help develop favorable attitudes toward health care information technology. Stress related to the use of new technology and the general impression that a new tool is going to reduce time available for bedside care contribute to nurses’ negative attitudes toward technology [8]. According to Lee [9], success with the implementation of technology is also related to the individual’s decision to adopt it or not. Nurses’ acceptance of computerized documentation was influenced by their perception of its advantages, whether they could see when it was being used by others, the complexity of the system, and the compatibility of its use with their values and experience [10].

### Theoretical Background

Numerous theories have been proposed that may help to explain the mecanisms involved in the adoption of a new behavior. Ajzen’s [11] theory of planned behavior (TPB) has been succesfully applied to study a range of behaviors in health care professionnals. In their systematic review, Godin et al demonstrated that the TPB explained 59% of the explained variance of intention [12]. According to Ajzen, the adoption of a new behavior is predicted by the person’s intention to engage in that behavior. In turn, intention depends on three main determinants: attitudes, subjective norms, and perceived behavioral control. Attitudes can be represented by the sum of the advantages and disadvantages related to adoption of the new behavior. Subjective norms consist of the person’s internalization of the reference groups’ opinions about the realization of the behavior. Finally, perceived behavioral control relates to the person’s impression that he or she has the required resources and capacities to adopt the new behavior. Some authors (eg, Gagnon et al [13]; Daneault et al [14]) include an additional dimension in Ajzen’s model to adapt it to health care workers: the professional norm. This dimension is related to a person’s integration of the specific normative pressures of one’s professional group. Ajzen also identifies three types of beliefs that may influence behavioral determinants: behavioral, normative, and control-related beliefs. The salient modal beliefs are the most common beliefs reported in a specific group of people concerning the adoption of a certain behavior. Consequently, it is imperative to identify those salient modal beliefs in the specific population under study in order to understand the factors explaining the intention to adopt a new behavior in a particular situation.

Shoham and Gonen [6] evaluated the intention of 411 nurses to use a computer at work, based on the TPB. In their study, 72% of nurses demonstrated a positive attitude toward the use of a computer at work. They also found that the department’s work environment, nurses’ work experience, stress generated by the use of a new computerized tool, and self-perception of computer skills influenced nurses’ intention to use a computer. This study included nurses who generally worked in a department other than emergency and evaluated nurses’ intention to use a computer at work rather than their intention to use specialized software.

A study of primary care nurses’ intention to use an electronic health record conducted in Quebec, Canada [15], found that the TPB explained 58% of the variance in intention. The main determinants of nurses’ intention to use the electronic record were normative beliefs, attitude, and perceived behavioral control.

Our review of the literature found no other study investigating the determinants of nurses’ adoption of computerized systems based on the TPB in a resuscitation situation. Also, there exist no questionnaires that evaluate the intention of nurses working in a resuscitation unit to use a computerized platform to chart critical patient information during care.

### Objectives

The aim of this study was thus to apply Ajzen’s theory to develop and validate a questionnaire that evaluates nurses’ intention to use an electronic medical charting system that includes a clinical decision support tool for the care of trauma patients in the resuscitation room.

## Methods

### Participants

All nurses working in the resuscitation unit of the emergency department of a tertiary trauma center in Quebec City, Canada, were identified by the chief nurse. The sample of this study comprised 87 nurses working in the resuscitation unit. We excluded nurses who were absent from their usual working shifts (eg, on vacation or absent for medical problems) during the recruitment period.

### Focus Groups

We held three focus groups to identify the salient modal beliefs of nurses working in the resuscitation unit in relation to the use of a new computerized platform for trauma care. Overall, 12 nurses participated (4 in each group). At the beginning of each focus group, the technician who had developed the software presented the key concepts and functions of the new platform. Then, 6 open questions were asked to identify their beliefs about adopting this system: (1 and 2) What would be the advantages or disadvantages of your use of the platform for trauma patient care in the resuscitation unit?, (3 and 4) What groups could approve or disapprove of your use of the platform in the resuscitation unit?, and (5 and 6) What would you consider to be a facilitator or an obstacle to the routine use of the platform for trauma patient care in the resuscitation unit? Each focus group lasted about 60 minutes and was audio recorded. Each participant received a Can $50 compensation.

### Questionnaire Design

Two investigators individually reviewed the content of each focus group with a standardized data extraction form and identified the most common themes reported by the participants. We then pooled the results and kept themes that had been reported at least 3 times (representing 25% of the participants) as the salient modal beliefs. A questionnaire (see Multimedia Appendix 1) was developed in which each of these salient modal beliefs was used as an item to evaluate the TPB constructs. We included 30 items in total: 8 to evaluate the advantages and disadvantages related to platform use (attitude), 6 to evaluate the people or groups that would approve or disapprove of use of the platform (social norm), 10 to evaluate the factors that could facilitate or limit platform use (perceived control), 3 to evaluate the intention to use the platform, and 3 to evaluate the professional norm. Each of these questions (question numbers 4 to 33 of the questionnaire) were assessed using a 7-point Likert scale (eg, total disagreement = 1, strong disagreement = 2, partial disagreement = 3, neutral = 4, partial agreement = 5, strong agreement = 6, total agreement = 7). We also included 13 questions to evaluate the nurses’ computer literacy in order to consider this variable in our analyses. These questions were translated and adapted (with the authors’ permission) from a validated questionnaire developed by Gassert and McDowell [16]. We used the same answer choices as those that were initially developed by Gasser and McDowell. We also included 3 sociodemographic questions in the questionnaire. The final questionnaire totaled 46 closed questions. The questionnaire was face validated by 3 research nurses, and we used their comments to improve the final version of the questionnaire (see Multimedia Appendix 1).

A cover letter was attached to the questionnaire to present the study’s objectives and a brief overview of the key characteristics of the platform. A notification at the bottom of the first page clearly indicated that returning the completed questionnaire signified consent to participate in the study. This study was approved by the hospital’s ethics review board.

### Data Collection

Participants were recruited in the emergency department between April 25 and May 6, 2011. All the nurses working in the resuscitation unit and present during their regular work shifts were individually contacted by the principal investigator or a research assistant. Each questionnaire was identified by a unique identification number. Participants were instructed to return their completed questionnaire to an identified secure box. Participants who accepted to complete the questionnaire were invited to complete it a second time 2 weeks later to measure the questionnaire’s reliability. Participants received a Can $5 gift card for each questionnaire completed (one for phase 1 and one for phase 2).

### Statistical Analyses

First, we assessed the temporal stability of the measurement of theoretical constructs in the questionnaire by the intraclass correlation coefficient (ICC) for answers to the first and second questionnaires (test-retest reliability) [17-19]. We considered test-retest reliability to be fair for values of ICC between .4 and .59, good for values between .60 and .74, and excellent for values higher than .75 [20-22]. Then, we calculated Cronbach alpha coefficients to assess the internal consistency of the theoretical constructs [17-19]. We explored construct validity by examining Spearman correlations between items and theoretical constructs. Correlations between items forming the same construct were also explored to detect collinearity problems.

Second, we examined descriptive analyses of sociodemographic characteristics and theoretical variables. Associations between these variables were explored with chi-square tests for the univariate analysis, and a logistic regression was performed to identify the determinants of nurses’ high versus moderate intention to use the platform. Using the median intention score as the cut-off point, we defined high intention as a score of >6 (total agreement) on the Likert scale and moderate intention as a score ≤6. All analyses were done with SAS version 9.2 (SAS Institute, Cary, NC, USA).

## Results

In phase 1, we received 62 of the 70 questionnaires administered (89% response rate). Of the 27 questionnaires administered in the second phase (2 weeks later), 25 were completed (93% response rate). We chose to administer 27 questionnaires in the second phase to obtain results for the retest phase from at least 30% of our initial sample. This number corresponds to the number of participants recommended for calculating temporal stability in a psychosocial questionnaire based on the TPB [23]. Descriptive statistics of the sample are presented in Table 1. More women than men participated in the study, which is consistent with the gender distribution in the population. Of the respondents, 66% (40/61) were 40 years of age or less and 30% (18/61) had more than 15 years of experience. As the study questionnaire was entirely anonymous, it was not possible to identify nurses who did not return their questionnaire. However, to assess the possibility of nonresponse bias, we compared respondents’ characteristics with those of Quebec nurses [24]. The proportion of nurses aged less than 40 years and the proportion of men in our sample were higher than those of Quebec nurses. These differences can be explained by the fact that nurses from our sample work exclusively in the emergency department, and for numerous reasons (eg, shift work, stress), emergency department nurses tend to be younger, and there is a higher proportion of men.

**Table 1:** Demographic characteristics of respondents and high versus low intention to use the platform.

Characteristic of sample (n = 62)		Frequency	χ^2 ^(high versus low intention to use the platform)^a^	*df*	*P *value
**Gender** ^b^			1.3	1	.26
	Male	16 (26%)		
	Female	45 (74%)		
**Age (years)** ^b^			0.9	2	.63
	<31	21 (34%)		
	31–40	19 (31%)		
	>40	21 (34%)		
**Experience (years)**			3.5	3	.32
	<5	17 (27%)		
	6–10	13 (21%)		
	11–15	14 (23%)		
	>15	18 (29%)		

^a ^High intention: score of >6 on Likert scale; low intention: score of ≤6 on Likert scale.

^b ^n = 61.

### Internal Consistency

Table 2 shows the Cronbach alpha coefficients for each construct of the questionnaire. All constructs had a Cronbach alpha higher than .7, which is considered excellent [25,26].

**Table 2:** Internal consistency of the constructs.

Construct	Number of items	Cronbach alpha	95% CI^a^
Attitude	8	.85	.77–.90
Perceived control	10	.82	.52–.89
Professional norm	3	.84	.71–.93
Social norm	6	.85	.80–.90
Intention	3	.94	.80–.99
Computer literacy	13	.89	.85–.92

^a ^Confidence interval.

### Reliability

Table 3 shows ICC results for each construct. Temporal stability was shown to be good for the professional norm and social norm constructs and fair for the other constructs.

**Table 3:** Test–retest stability of constructs (n = 25).

Construct	ICC^a^	95% CI^b^
Attitude	.41	.03–.69
Perceived control	.41	.04–.69
Professional norm	.66	.37–.83
Social norm	.60	.28–.80
Intention	.59	.27–.80

^a ^Intraclass correlation coefficient.

^b ^Confidence interval.

### Descriptive Statistics for the Constructs

For each construct, we evaluated the mean score of nurses’ responses on the 7-point Likert scale (Table 4). Overall, the participants had a high intention to use the computerized platform when it becomes available (mean 6.35 out of a maximum of 7).

**Table 4:** Means and standard deviations of constructs.

	Attitude	Perceived control	Professional norm	Social norm	Intention	Computer literacy^a^
Mean	5.81	5.94	6.10	5.63	6.35	2.58
SD	0.96	0.81	1.13	0.96	1.06	1.12

^a ^Score on a 4-point Likert scale.

### Bivariate Correlations Between Constructs

We explored correlations between constructs as shown in Table 5. According to Kline [27], multicollinearity is present when the correlation between two independent variables is greater than .85. The coefficient of correlation between each construct was acceptable, which indicates that a multicollinearity problem was not present.

The mean score was 2.58 (out of a maximum of 4) for computer literacy. This corresponds to some experience with the various computer software packages or devices evaluated (Table 6). The internal consistency for computer literacy was excellent (Cronbach alpha = .89, 95% confidence interval [CI] .85–.92). Temporal stability was acceptable with an ICC of .68 (95% CI .40–.84). A large majority (59/62, 95%) of respondents indicated that they had their own computer at home. The nurses indicated that they had less experience with two types of software: databases (eg, Microsoft Access) and computerized statistical analysis software (eg, SAS, SPSS, Maple).

**Table 5:** Spearman correlation coefficients (and *P *values) between each construct pair (n = 62).

Construct	Intention	Attitude	Perceived control	Professional norm	Social norm	Computer literacy
Intention	1.00	.42 (<.001)	.35 (.01)	.62 (<.001)	.31 (.02)	.30 (.02)
Attitude		1.00	.38 (.003)	.39 (.002)	.43 (<.001)	.38 (.002)
Perceived control			1.00	.35 (.01)	.38 (.003)	.19 (.14)
Professional norm				1.00	.36 (.004)	.19 (.14)
Social norm					1.00	.26 (.04)
Computer literacy						1.00

**Table 6:** Assessment of computer literacy in the study sample^a^.

Computer skill	Score	
	Mean	SD
Microcomputer use (PC or Mac)	2.97	0.85
Keyboard/typing skills	3.42	0.64
Word processing	2.98	0.98
Spreadsheet (eg, Microsoft Excel)	2.16	0.93
Database (eg, Microsoft Access)	1.95	0.86
Email	3.52	0.62
Internet	3.61	0.52
Bibliographic database searching (eg, CINAHL, Medline, PubMed)	2.02	0.98
Computerized statistical programs	1.37	0.61
Presentation software (eg, PowerPoint)	2.08	1.00
Personal digital assistant (eg, Palm, iPod, iPhone)	2.50	1.22
Use of cell phone with Web capability	2.42	1.14
Do you have your own personal computer at home (yes), n (%)	59/62	95%

^a ^Adapted from Gassert and McDowell [16] with their permission.

### Logistic Regression Model

We tested a logistic regression model with the full sample (n = 62) to identify the main determinants of nurses’ low versus high intention to use the platform. The only construct that predicted the nurses’ intention to adopt the computerized platform was the professional norm (odds ratio 3.31, 95% CI 1.41–7.78; see Table 7). The Nagelkerke *R* *2 *obtained by this model is .41.

**Table 7:** Logistic regression model for dichotomized intention construct (high vs low intention)^a^.

Construct	Estimated (β)	OR^b^	95% CI^c^	*P *value
Attitude	0.511	1.67	0.65–4.30	.29
Perceived control	0.266	1.31	0.44–3.89	.63
Professional norm	1.197	3.31	1.41–7.78	.01
Social norm	0.022	1.02	0.49–2.15	.95
Computer literacy	0.628	1.87	0.60–5.89	.28

^a ^Regression model equation: logit[probability((score intention >6) = 1)] = β_0 _+ β_1_.attitude + β_2_.perceived control + β_3_.professional norm + β_4_.social norm + β_5_.computer skills.

^b ^Odds ratio.

^c ^Confidence interval.

## Discussion

To the best of our knowledge, this is the first study that applies the TPB to assess nurses’ intention to use a computerized platform as a support to resuscitation work in an emergency department. We believe this computerized platform is an important innovation because it is an electronic medical record and a clinical decision support system created for nurses working in the resuscitation unit. We developed a questionnaire based on the TPB that showed good internal consistency and fair temporal stability. Furthermore, we translated into French and validated a 13-question instrument developed by Gassert and McDowell [16] to evaluate nurses’ computer literacy. Because the response rate was high (89%), it is unlikely our study was subject to a self-selection bias.

Of particular interest is that only the professional norm significantly influenced the intention to use the platform in our logistic regression model. This can be partially explained by the fact that in the year preceding the present study, the emergency department completed a health services quality accreditation process, conducted by Accreditation Canada. The accreditation of the emergency department was based on five key elements. The maintenance of accessible and efficient clinical information systems, and the creation of prepared and proactive teams were two of these five principles. Participation in this accreditation process may have sensitized nursing personnel to current professional norms.

From the literature, it could be expected that nurses with higher computer literacy would be more likely to have a higher intention to use the platform [7]. Nurses in our study evaluated themselves as having some to moderate computer skills (58/62, 94%) with a mean score of 2 or 3), and 95% (59/62) claimed to have a personal computer at home. However, in our study we only found a trend toward an association (odds ratio 1.87, 95% CI 0.60-5.89). Our regression model indicated that the TPB variables explained 41% of the variance in nurses’ intention to use the computerized platform, which is comparable with what is reported in the TPB literature [12].

Age, gender, and number of years of experience did not have any influence on nurses’ intention to use the platform. These results are different from those reported in previous studies [7,28,29]. Previous studies about nurses’ adoption of computerized information systems have shown that perceived behavioral control, normative beliefs, and attitudes influenced their intention to adopt an electronic health record [15]. Perceived behavioral control and attitude were also related to nurses’ intention to use a computer in a hospital setting in the study by Shoham and Gonen [6]. The lack of impact of these determinants in our study may be related to the small number of participants and the fact that intention was very high among them. Thus, if most nursing staff already support the adoption of the platform, the intention of nurses is less likely to be influenced by social pressures. The same explanation can be provided for the attitudinal beliefs: everyone shares the same beliefs about the advantages of using the computerized information system in the resuscitation unit, so attitude is not likely to have an impact on nurses’ intention. The lack of influence of the behavioral control beliefs could indicate that the perceived facilitators for use of the platform were very similar among the respondents. The fact that this platform was developed in the same resuscitation unit by clinicians who already work with them may have led nurses to perceive few barriers to their adoption of the platform. Furthermore, it is possible that they felt some kind of peer pressure to answer positively to the questionnaire even though the questionnaires were answered anonymously. Indeed, it could be useful to administer this questionnaire in other centers not involved in the development of this electronic platform.

As participants during phase 1 were aware that there would be a second phase to evaluate temporal stability, it is possible that the participants tried to remember their answers in phase 1 in order to write the same answers in phase 2. We believe it is unlikely that this bias influenced the results because phase 1 and 2 were separated by 2 weeks and because the questionnaire included 46 questions.

Some limitations should be noted in relation to our results. First, our population was small, which explains our small sample size, even though we had a good response rate. Furthermore, our study was conducted in only one emergency department. Generalization of the results to other populations and settings is not possible, but the instrument that was developed can be applied in other similar settings. Second, clinicians who worked in the same emergency department participated in the development of the software. This could have influenced the results by introducing a social desirability bias. Third, nurses’ intention to use the computerized platform was high (mean of 6.35 out of 7), thus leading to very low variance in the intention scores, which could have limited the performance of the theoretical model. Fourth, for the perceived control construct, 2 items of the questionnaire did not correlate well with the other items of the construct. These 2 items were (1) the fact that some people are resistant to change, and 2) the eventual presence of computer bugs. These were the only two obstacles we identified in the focus groups. It was not possible to create a new construct with those 2 items because it would have been in contradiction to experts’ recommendations in relation to measurement of psychosocial constructs [23,30]. We decided to keep these items in the questionnaire because we felt they may have an impact on nurses’ intention to use the platform, but the results indicated that it this was not the case. Finally, because our questionnaire contained 46 questions, our results may have been influenced by respondent fatigue (the weariness effect).

### Conclusion

This study evaluated nurses’ intention to use a computerized platform in the resuscitation unit of an emergency department. The respondents’ intention was very high, which suggests that the implementation of this innovation is likely to be successful in our emergency department. Once it is implemented, it will be interesting to assess whether this high intention has translated into a high adoption rate of the computerized platform. The questionnaire developed proved to have good internal consistency and good temporal stability. The platform developers hope to implement this computerized platform in other emergency departments in Quebec. The validated questionnaire could be used in other emergency departments prior to implementation of the computerized platform to orient the actions of managers toward improving the likelihood of a successful implementation. Evaluation of this questionnaire in other trauma centers with different settings could help to assess its validity and could help to identify the potential barriers and facilitators to implementation of this novel charting and decision support platform in other trauma centers in the province.

## Multimedia Appendix 1

The Questionnaire developed to evaluate nurses' intention to use a computerized platform in the resuscitation unit.

## Abbreviations

CI: confidence interval
ICC: intraclass correlation coefficient
TPB: theory of planned behavior
