# Supplementary material for: Exploring Nurses’ Intention to Use a Computerized Platform in the Resuscitation Unit: Development and Validation of a Questionnaire Based on the Theory of Planned Behavior
Source: Interact J Med Res. 2012 Sep 13;1(2):e5. doi: 10.2196/ijmr.2150 (PMC3626134; doi:10.2196/ijmr.2150)
Supplement: Supplementary file 1 [file ijmr_v1i5e5_app1.pdf]

Questionnaire # _____

Questionnaire addressed to nurses working in the reanimation unit.

Instructions:

1. You must be a nurse working in the reanimation unit in order to complete the following questionnaire.
2. Once completed, please deposit de the questionnaire in the lockedsecured box located in your rest area.

In order to help us validate the questionnaire, do you agree to complete the same questionnaire in two weeks? ☐Yes ☐No

Section 1 General information:

1. Which age category do you fall into?

≤20 years old	21-30 years old	31-40 years old	41-50 years old	51-60 years old	61-70 years old	71-80 years old

2. What is your gender?

	male

3. For how long have you worked as a nurse?

≤1 year	2-5 years	6-10 years	11-15 years	16-20 years	21-25 years	□26 years

Section 2 The computerized platform

During the comingnext year, if I used the computerized platform for each trauma cases in the resuscitation unit…

4. Iit would allow me to haveget quick access to crucial informations about my patient.

Totally Disagree	Strongly Disagree	Somewhat Disagree	Neutral	Somewhat Agree	Strongly Agree	Totally Agree

5. Iit would allow me to organizecollegiate medical informations more clearly.

Totally Disagree	Strongly Disagree	Somewhat Disagree	Neutral	Somewhat Agree	Strongly Agree	Totally Agree

6. Iit would allow me to organizecolligate medical informations more precisely.

Totally Disagree	Strongly Disagree	Somewhat Disagree	Neutral	Somewhat Agree	Strongly Agree	Totally Agree

7. Iit would allow me to createcolligate more complete medical records which are more complete.

Totally Disagree	Strongly Disagree	Somewhat Disagree	Neutral	Somewhat Agree	Strongly Agree	Totally Agree

8. Iit would allow me to more easily identify nurses who took care of a patient.

Totally Disagree	Strongly Disagree	Somewhat Disagree	Neutral	Somewhat Agree	Strongly Agree	Totally Agree

9. Iit would generate another source of stress for me in the resuscitation unit.

Totally Disagree	Strongly Disagree	Somewhat Disagree	Neutral	Somewhat Agree	Strongly Agree	Totally Agree

10. Iit could force me to stop working in the resuscitation unit.

Totally Disagree	Strongly Disagree	Somewhat Disagree	Neutral	Somewhat Agree	Strongly Agree	Totally Agree

If all nurses working in the reanimation unit used the computerized platform for trauma patients in the reanimation unit…

11. Iit would improve the general effectivenessglobal efficacy of nurses working in the reanimation unit.

Totally Disagree	Strongly Disagree	Somewhat Disagree	Neutral	Somewhat Agree	Strongly Agree	Totally Agree

The following persons would approve/disapprove of my eventual use of the computerized platform in the reanimation unit:

12. Nurses with less computer experience.

Would strongly approve	Would moderately approve	Would slightly approve	Neutral	Would slightly disapprove	Would moderately disapprove	Would strongly disapprove

13. Emergency physicians.

Would strongly approve	Would moderately approve	Would slightly approve	Neutral	Would slightly disapprove	Would moderately disapprove	Would strongly disapprove

14. Patients.

Would strongly approve	Would moderately approve	Would slightly approve	Neutral	Would slightly disapprove	Would moderately disapprove	Would strongly disapprove

15. The Quebec's Order of Nurses.

Would strongly approve	Would moderately approve	Would slightly approve	Neutral	Would slightly disapprove	Would moderately disapprove	Would strongly disapprove

16. Administrative hospital personnel.

Would strongly approve	Would moderately approve	Would slightly approve	Neutral	Would slightly disapprove	Would moderately disapprove	Would strongly disapprove

17. Accreditationgreement Canada.

Would strongly approve	Would moderately approve	Would slightly approve	Neutral	Would slightly disapprove	Would moderately disapprove	Would strongly disapprove

To have a qQuick access to the software with a magnetic card would facilitate my use of the platform.

Totally Disagree	Strongly Disagree	Somewhat Disagree	Neutral	Somewhat Agree	Strongly Agree	Totally Agree

18. The use ofidea of using a tactile screen to enter information on the computerized platform would facilitate my work in the resuscitation unit.

Totally Disagree	Strongly Disagree	Somewhat Disagree	Neutral	Somewhat Agree	Strongly Agree	Totally Agree

19. The fact that all the medical softwares used in the resuscitation unit would be on the same computer (eg : Cristal-Net, Rea Scribe software, mMedical dDatabases…) in the resuscitation unit would facilitate my use of the computerized platform.

Totally Disagree	Strongly Disagree	Somewhat Disagree	Neutral	Somewhat Agree	Strongly Agree	Totally Agree

20. The fasterst the software will be, the more likely it will be that I will use it in the resuscitation unit.

Totally Disagree	Strongly Disagree	Somewhat Disagree	Neutral	Somewhat Agree	Strongly Agree	Totally Agree

21. Completing training abouton the computerized platform, prior to its implementation, would facilitate my use of it.

Totally Disagree	Strongly Disagree	Somewhat Disagree	Neutral	Somewhat Agree	Strongly Agree	Totally Agree

To have the opportunityIf I were able to trymanipulate the software of the platform before its implementation, this would facilitate my use of it.

Totally Disagree	Strongly Disagree	Somewhat Disagree	Neutral	Somewhat Agree	Strongly Agree	Totally Agree

22. If I hadreceive support from an expert nurse colleague duringfor my first shifts working with the platform, this it would help me use it.

Totally Disagree	Strongly Disagree	Somewhat Disagree	Neutral	Somewhat Agree	Strongly Agree	Totally Agree

23. If The idea that a nurse colleague nurse will were to be involved in the improvement process of the platform once implemented, this would facilitate my use if it.

Totally Disagree	Strongly Disagree	Somewhat Disagree	Neutral	Somewhat Agree	Strongly Agree	Totally Agree

24. For me, the fact that some peoplersons are against changes could be an obstacle to my use of the platform.

Totally Disagree	Strongly Disagree	Somewhat Disagree	Neutral	Somewhat Agree	Strongly Agree	Totally Agree

25. If there are The eventual presence of computerinformatic buogs, this could be an obstacle to my use of the platform in the resuscitation unit.

Totally Disagree	Strongly Disagree	Somewhat Disagree	Neutral	Somewhat Agree	Strongly Agree	Totally Agree

26. I intend to use When the platform when it becomes is available in the resuscitation unit., I have the intention to use it.

Totally Disagree	Strongly Disagree	Somewhat Disagree	Neutral	Somewhat Agree	Strongly Agree	Totally Agree

27. The oddslikelihood that I will use the platform (when available) in the reanimation unit are high.

Totally Disagree	Strongly Disagree	Somewhat Disagree	Neutral	Somewhat Agree	Strongly Agree	Totally Agree

28. If the opportunity presents itselfshows up, I will use the platform in the resuscitation unit.

Totally Disagree	Strongly Disagree	Somewhat Disagree	Neutral	Somewhat Agree	Strongly Agree	Totally Agree

29. In the resuscitation unit, it is expected that nurses will use the platform when available.

Totally Disagree	Strongly Disagree	Somewhat Disagree	Neutral	Somewhat Agree	Strongly Agree	Totally Agree

30. In my hospital, the nurses working in the resuscitation unit will have to use the platform when it becomesis available.

Totally Disagree	Strongly Disagree	Somewhat Disagree	Neutral	Somewhat Agree	Strongly Agree	Totally Agree

31. A competent nurse should use the platform in the resuscitation unit when it becomesis available.

Totally Disagree	Strongly Disagree	Somewhat Disagree	Neutral	Somewhat Agree	Strongly Agree	Totally Agree

Section 3 Questions on computer experience :

Please circle the number under the word that best describes your level of experience with the following computer hardware or software.

NONE = I have never used this type of computer or software before.

SOME = I have used this computer or software but cannot use it by myselfindependently.

MODERATE = I use this computer or software, but occasionally need assistance.

EXTENSIVE = I use this computer or software frequently and could show others how to use it

None Some Moderate Extensive

34. Microcomputer (PC or Mac)	1	2	3	4
35. Keyboard/typing skills	1	2	3	4
36. Word processing (Microsoft Word)	1	2	3	4
37. Spreadsheet (Example: Microsoft Excel)	1	2	3	4
38. Data Base (Example: Microsoft Access)	1	2	3	4
39. Electronic mail (E-mail)	1	2	3	4
40. Internet/World Wide Web	1	2	3	4
41. Bibliographic database searching (CINAHL, Medline, Pubmed, etc)	1	2	3	4
42. Computerized statistical analysis (SAS, SPSS, MAPLE, NCSS, etc)	1	2	3	4
43. Presentation graphics (such as PowerPoint, ToolBook, etc)	1	2	3	4
44. Personal Digital Assistant (Palm Pilot, Ipod touch, Iphone, etc)	1	2	3	4

45. Use of Cell Phone with Web Capability 1 2 3 4

46. Do you have aown your personal 1 2 3
4

computer at home.

Thank you for your participation!
